# Immunoglobulin A (IgA) Nephropathy: A Clinicopathologic Study in a Tertiary Care Center in Saudi Arabia

**DOI:** 10.7759/cureus.19445

**Published:** 2021-11-10

**Authors:** Omar A Bokhary, Hanadi M Alhozali, Maha K Alghamdi, Ahmed M Abulfaraj, Abdulaziz S Alkhallagi, Abdulmohsen S Aldharrab, Faisal S Alyahya, Reem A Al Zahrani

**Affiliations:** 1 Medicine, King Abdulaziz University, Jeddah, SAU; 2 Nephrology, King Abdulaziz University Hospital, Jeddah, SAU; 3 Pathology, King Abdulaziz University, Jeddah, SAU

**Keywords:** retrospective studies, gender role, proteinuria, haematuria, iga nephropathy

## Abstract

Background: Immunoglobulin A nephropathy (IgAN) is the most common primary aetiology of glomerulonephritis worldwide, and it is the most important type in terms of morbidity and mortality. IgAN involves the deposition of immune bodies in the mesangial cells of the kidney, which causes renal glomerular damage, haematuria, proteinuria, and various other symptoms. Previous studies have mainly focused on the East Asian population, with few studies conducted in Saudi Arabia, particularly in the western region. The diagnosis requires a biopsy, which should be examined by an expert and classified according to the Oxford classification system.

Objectives: Analyze the clinical, pathological, and laboratory features of male and female patients diagnosed with IgAN at King Abdulaziz University Hospital (KAUH).

Methods and materials: This was a retrospective record review conducted at KAUH in Jeddah, Saudi Arabia between May-June 2021. The study included 18 patients diagnosed with IgAN by biopsy, and their clinical, laboratory and pathological data were evaluated and classified according to the Oxford classification system.

Results: Demographic data revealed a male predominance of 66.7%. The most common pathological finding was mesangial proliferation, and the most common presentation was haematuria. For treatment options, corticosteroids were the most prescribed drugs. A significant relationship was found between IgAN with increased serum creatinine and male sex (P = 0.017). Additionally, a significant relationship was observed between decreased estimated glomerular filtration rate (eGFR) in IgAN and the male sex (P = 0.006).

Conclusions: We found a difference in terms of pathological, clinical and laboratory presentations of IgAN between males and females. Men generally had worse kidney function at presentation and advanced Oxford classification in their kidney biopsies compared to women.

## Introduction

Immunoglobulin A nephropathy (IgAN), often referred to as Berger's disease, is an autoimmune glomerulonephritis, with a predominance of IgA deposits in the glomerular mesangium, either alone or in combination with IgG, IgM, or both [1-3]. It is considered the most prevalent form of nephritis worldwide. Its prevalence has increased significantly in Saudi Arabia, and it is one of the primary causes of chronic kidney disease (CKD) [4,5]. Although multiple studies have attempted to illustrate the aetiology of IgAN, the results have been conflicting. Many researchers have reported that a combination of genetic, environmental, and geographic factors plays a role in the pathogenesis of IgAN [6,7]. The clinical presentation is variable, but it usually presents as repeated episodes of haematuria with or without proteinuria, and as a consequence, these patients develop hypertension [8]. Due to the wide range of clinical presentations, glomerular diseases necessitate renal biopsy. The characteristics of IgAN on light microscopy can vary extensively between patients and within a single biopsy sample. Typically, expansion of the mesangial matrix and hypercellularity is seen; other possible glomerular lesions include segmental scarring, focal necrosis, and crescents in the Bowman’s capsule [3].

A study conducted by the International IgA Nephropathy Network and the Renal Pathology Society reported that pathologists had classified their patients' renal biopsies and taken into account their clinical data to have a reference set of pathological variables to be used as indicators for renal survival [4]. The patients were scored using, what is referred to as the Oxford classification, a four-component MEST score which explores the presence of mesangial hypercellularity (M), endocapillary hypercellularity (E), segmental glomerulosclerosis (S), and tubular atrophy/interstitial fibrosis (T). A 2016 study stated that integrating the MEST score with clinical data of the patients had the same predictive ability as monitoring the clinical data for two years [9]. Many attempts have been made to analyse the clinicopathologic correlation in patients with IgAN. A study conducted in Iran revealed that male patients had a higher MEST score on biopsy [10]. Multiple studies conducted in China reported that male patients presented with more advanced clinical and pathological features than females [11,12]. Many studies reported macroscopic haematuria as the hallmark of IgA nephropathy, and other presentations of the disease might occur more commonly in adults, including hypertension and CKD [3,13]. A previous study conducted in Saudi Arabia in 2010 explored the clinical and pathological characteristics of IgAN and classified the patients according to the Haas classification, concluding that the most common presenting symptom was haematuria, followed by proteinuria [14]. Another study conducted in China in 2015 concluded that benign clinical presentation did not necessarily reflect the severity of histological renal damage; a patient might present with minimal symptoms but could have a significant renal injury [15]. Previous studies in our region focused on outdated histological classifications, and the characteristics of IgAN using the updated Oxford classification have not been investigated [14]. Therefore, the aim of this study is to analyse the clinical, pathological, and laboratory features of IgAN in male and female patients at King Abdulaziz University Hospital (KAUH) and classify the disease according to the Oxford classification.

## Materials and methods

This single-center retrospective record review study was conducted at KAUH, a tertiary care centre in Jeddah, Saudi Arabia.

This research was authorized by the Institutional Review Board of KAUH (Ref: 38-21). The procedures followed were in accordance with the ethical standards of the responsible committee based on the Good Clinical Practice Guidelines. Informed consent was waived due to the retrospective nature of the study. We collected and analysed the data of 67 patients diagnosed with primary IgAN by biopsy between 1989 and 2021. The number of patients who fulfilled the inclusion criteria was 18, and we included patients of all ages diagnosed with IgAN by biopsy. Patients with no documentation of laboratory investigations, secondary IgAN due to other diseases such as Henoch Schonlein purpura, and those with established end-stage renal disease (ESRD) at the time of the first diagnostic biopsy were excluded.

Clinical data

The clinical data of all patients diagnosed with IgAN on biopsy was acquired through a review of the hospital records. Data retrieved included information about gender, nationality, age, weight (Kg), height (cm), and first clinical presentation, including the presence of nephrotic syndrome, isolated haematuria, isolated proteinuria, acute kidney injury, and hypertension. The following concomitant morbidities were also considered: diabetes, ischemic heart disease, chronic heart failure, malignancy, cerebrovascular disease, autoimmune disease, thyroid disease, chronic liver disease, peripheral vascular disease, respiratory disease, and CKD. Blood pressure (BP), blood urea nitrogen (BUN), total cholesterol, triglycerides, and microhaematuria, defined as the presence of >3 red blood cells per high power field (HPF), serum creatinine (sCr) at the time of biopsy, haemoglobin level, proteinuria (g/dL) and estimated glomerular filtration rate (eGFR) (mL/min/1.73 m2). eGFR was measured using the Chronic Kidney Disease Epidemiology Collaboration (CKD-EPI) equation for adults [16], in which the normal range of eGFR is between 90 and 120 mL/min/1.73m2; for children, the bedside Schwartz equation was used [17]. Finally, information about the prescribed medication regimen at the time of biopsy was also retrieved and included (the medications comprised angiotensin converting enzyme inhibitors/angiotensin receptor blockers, corticosteroids, calcium channel blockers, loop diuretics, beta-blockers, statins, and immunosuppressive medications).

Pathological data

The results of each renal biopsy were classified by an expert pathologist using the updated Oxford classification: mesangial hypercellularity (M0/M1; M1 was defined as > 50% of the glomeruli showing four or more cells in one or more mesangial areas, not including the central core or region of the vascular pole); endocapillary hypercellularity (E0/E1; absent/present), segmental glomerulosclerosis (S0/S1; absent/present), tubular atrophy/interstitial fibrosis (T0/T1/T2;< 25%/25%-50%/> 50%, respectively), and cellular or fibrocellular crescents (C0/C1/C2; crescents absent or present in at least one glomerulus/in > 25% of the glomeruli) [18].

Statistical analysis

Microsoft Excel was used for data entry and the Statistical Package for Social Sciences (SPSS) version 21 (SPSS Inc., Chicago, IL) was used for statistical analysis. Mean and standard deviation (SD) were calculated to describe continuous variables, while frequencies and percentages were used for categorical variables. Student’s t-test and chi-square test were used to evaluate the differences between continuous and categorical variables, respectively. Statistical significance was set at P < 0.05.

## Results

Eighteen patients were enrolled in this study, of which 66.7% were men (Table 1). The mean age of the patients was 24 ± 14.2 years (range, 3-48 years) and the mean body mass index was 23.9 ± 4.32 kg/m2. The patients were categorised into three groups based on age, namely 3-16, 17-25, and 26-50 years. Approximately 38.9% of the patients were in the first group, 22.2% in the second group, and 38.9% in the third group. Saudis represented over half of the cohort (55.6%).

**Table 1:** Patient characteristics, clinical features, and comorbidities. Data are presented as n (%) unless otherwise specified. SD: standard deviation.

Variables	Frequency (Percent)*
Patient Demographics	
Males	12 (66.7)
Females	6 (33.3)
Age, yrs (mean ± SD)	24 ± 14.2
Weight, kg (mean ± SD)	69.7 ± 14.8
Height, cm (mean ± SD)	166.56 ± 8.52
Clinical Presentation	
Haematuria	8 (44.4)
Nephrotic syndrome	7 (38.9)
Hypertension	7 (38.9)
Isolated Proteinuria	4 (22.2)
Nephritic syndrome	1 (5.6)
Acute Kidney Injury	1 (5.6)
Comorbidities	
Respiratory disease	3 (16.7)
Thyroid disease	2 (11.1)
Chronic kidney disease	1 (5.6)
Malignancy	1 (5.6)
Vital parameters	
Systolic blood pressure (mean ± SD)	130 ± 28.3
Diastolic blood pressure (mean ± SD)	79.5 ± 19.1

The most common presenting symptoms were isolated haematuria (44.4%), nephrotic syndrome (38.9%), hypertension (38.9%), isolated proteinuria (22.2%), nephrotic syndrome (5.6%), and acute kidney injury (5.6%). The most prevalent concurrent morbidities were respiratory disease (16.7%), followed by thyroid disease (11.1%).

As shown in Table 2, corticosteroids were the most commonly used drugs for treatment (66.7%), followed by loop diuretics (55.6%).

**Table 2:** Prescription frequency of commonly prescribed drugs. ACE I: angiotensin-converting-enzyme inhibitors.

Variable	N (%)
Corticosteroids	12 (66.7)
Loop diuretics	10 (55.6)
ACE I	9 (50)
Calcium channel blocker	7 (38.9)
Beta blockers	5 (27.8)
Statins	4 (22.2)
Immunosuppressant	1 (5.6)
Statins	4 (22.2)

The laboratory findings are shown in Table 3. The mean serum creatinine was greater in men (150.7 ± 92.9 µmol/L versus 47.4 ± 189 µmol/L in women). Similarly, the mean eGFR in men was higher than in women (50.9 ± 31.4 mL/min/1.73m2 versus 113.2 ± 28.1 mL/min/1.73m2 in women). A significant relationship existed between the male sex and higher sCr levels (P = 0.003). Moreover, a significant relationship existed between the male sex and lower eGFR (P = 0.006). No other significant relationships were observed.

**Table 3:** Laboratory findings on the day of the biopsy. Cr: creatinine; eGFR: estimated glomerular filtration rate.

Variable	Male	Female	P-value
Serum Cr level (µmol/L)	150.7 ± 92.9	47.4 ± 18.9	0.003
Blood urea nitrogen (mmol/L)	11.6 ± 10.6	4.2 ± 0.9	0.115
Serum albumin (mmol/L)	23.3 ± 9.6	22.1 ± 9.8	0.980
Microscopic Hematuria (/HPF)	0.00	218.2 ± 226.8	0.150
Proteinuria (g/dL)	2.1 ± 1.1	1.7 ± 1.5	0.634
Hemoglobin level (g/dL)	13.2 ± 1.3	11.8 ± 1.6	0.069
Serum cholesterol (mmol/L)	513.8 ± 474.6	287.8 ± 274.8	0.479
Serum Triglycerides (mmol/L)	111.7 ± 129.4	306.5 ± 313.4	0.205
eGFR	50.9 ± 31.4	113.2 ± 28.1	0.006

**Figure 1:**
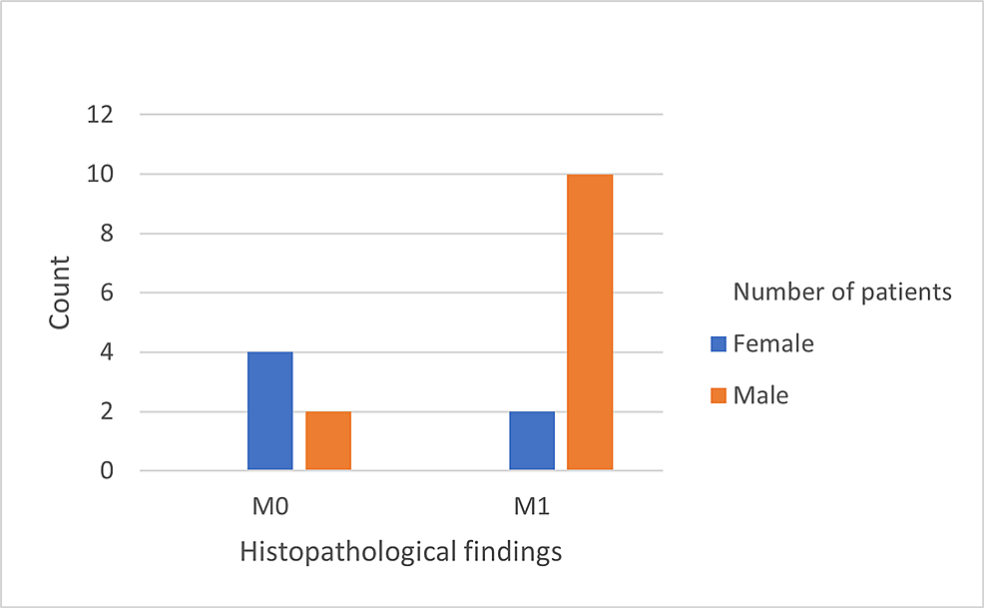
Frequency of mesangial hypercellularity findings in 18 cases diagnosed by biopsy.

**Figure 2:**
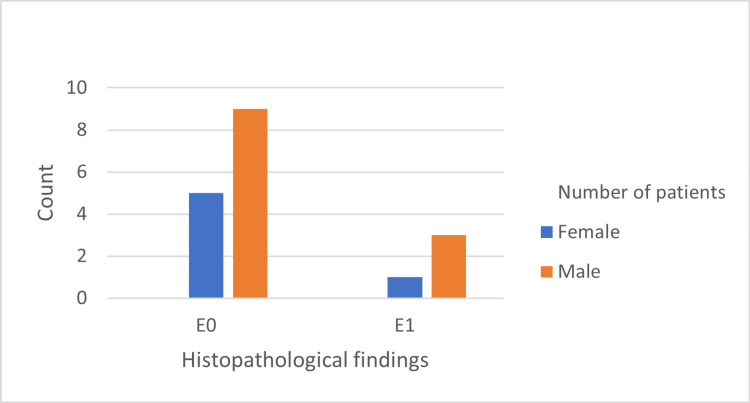
Frequency of endocapillary hypercellularity findings in 18 cases diagnosed by biopsy.

**Figure 3:**
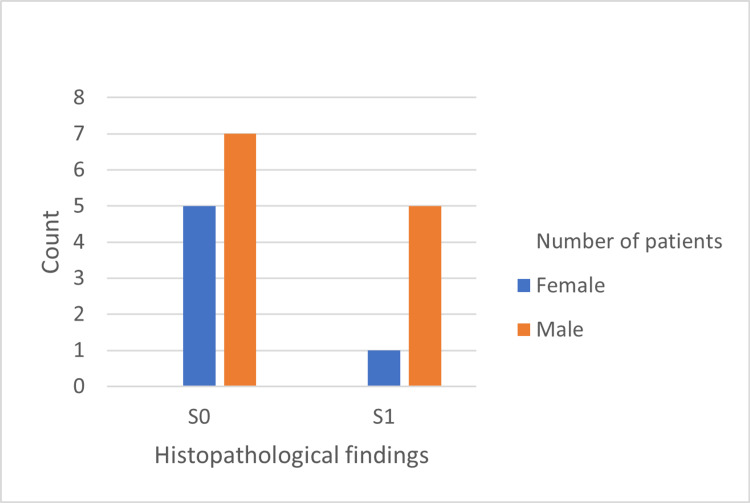
Frequency of segmental glomerulosclerosis findings in 18 cases diagnosed by biopsy.

**Figure 4:**
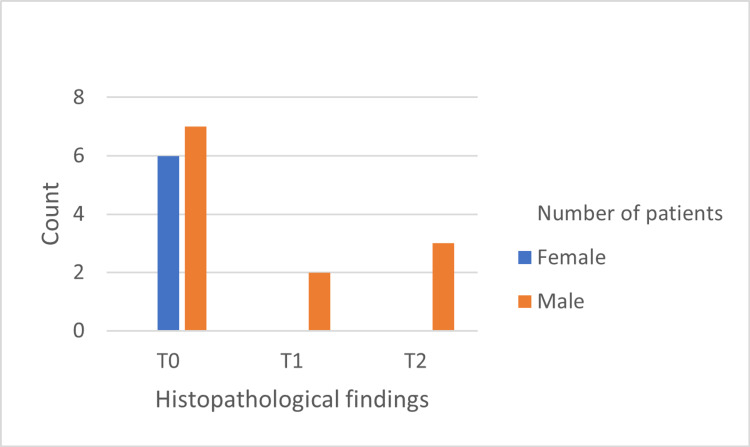
Frequency of tubular atrophy/interstitial fibrosis findings in 18 cases diagnosed by biopsy.

**Figure 5:**
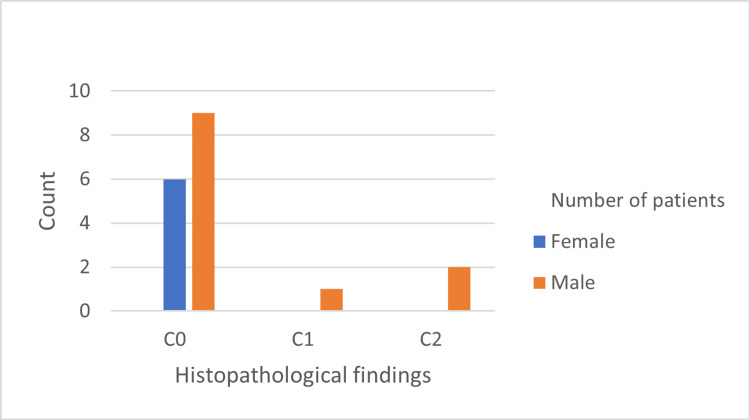
Frequency of tubular cellular/fibrocellular crescents findings in 18 cases diagnosed by biopsy.

The histopathological features are shown in Figures 1-5. The most common histopathological finding was mesangial proliferation, with 12 patients scoring M1; 10 of them were men. The segmental sclerosis score was S1 in six patients, of which five were men. The third most common histopathological finding was tubular atrophy/interstitial fibrosis with five patients scoring T1/T2, and all of them were men. No significant relationship was found between age, sex, and histopathological findings.

## Discussion

In our study, a significant relationship was observed between the male sex and increased serum creatinine levels (P = 0.017). This could be because men have a higher proportion of muscle mass and are generally more physically active, which in turn leads to increased muscle breakdown leading to an increased amount of creatinine produced in the body. Similar findings were reported in a study conducted in Brazil by Baxmann et al. [19], in which it was noted that physically active individuals tended to have higher serum creatinine than sedentary individuals. Moreover, there was a significant correlation between increased serum creatinine and body weight, particularly lean body mass. Additionally, in a study conducted in Canada, Cattran et al. reported that men tended to have lower creatinine clearance than women [20]. This suggests that male patients should have a higher reference range of normal serum creatinine to be considered abnormal than female patients.

Another sex-related difference was found in our study; males had lower eGFR than females, and a significant relationship between decreased eGFR and male sex was noted (P = 0.006). This is probably due to the protective effect of oestrogen on kidney function in females. Males, in general, are more prone to renal disease and tend to have a higher rate of progression of renal disease, possibly due to this phenomenon. Moreover, females produce lesser creatinine than males, which, in general, protects the kidneys from the increased workload. Several studies support these hypotheses. In a study conducted in China, Deng et al. reported that men were more likely than women to have a low eGFR [11]. Another study conducted in Estonia by Riispere et al. showed that IgA nephropathy advanced more rapidly in males than in females [21]. Moreover, the Modification of Diet in Renal Disease study found that women had a slower rate of eGFR decline and renal disease progression than men [22,23]. Finally, it was reported that the possible mechanisms underlying the renal protective role in the female sex seemed to be related to the effects of oestrogen [24]. These results should prompt clinicians to focus on IgAN disease progression in men. It is important to implement early screening programs in Saudi Arabia for the early diagnosis and control of IgAN in men and to treat these patients as early as possible to prevent worse clinical outcomes and rapid decline of renal function.

Haematuria was the most common presentation in our cohort, which could indicate that it is one of the hallmarks of IgAN. Among the two types of haematuria, macroscopic and microscopic haematuria, the former might be overlooked [8]. The presence of recurrent macroscopic haematuria or the detection of asymptomatic microscopic haematuria was found to be linked to a favourable prognosis [25,26]. The Estonia study conducted by Riispere et al. also reported that isolated haematuria was the most common presentation in patients with mild or early disease [21]. In a study conducted in Saudi Arabia by Khawajah et al., episodic haematuria and proteinuria were the most common presentations of the disease [14]. This is an important finding, and we strongly recommend early screening programs for IgAN to be initiated by health authorities in Saudi Arabia, with an emphasis on isolated haematuria as the most common presentation in the early stage of the disease. Hypertension and nephrotic syndrome were the second most common presentations in our study, indicating that these Clinical presentations are strongly related to IgAN. It has been reported that hypertension, proteinuria, renal dysfunction, and more severe renal pathological alterations are associated with renal prognosis in IgAN [12]. Deng et al. reported that proteinuria and hypertension were important risk factors for IgAN. Additionally, they reported proteinuria as an important predictor of disease progression [11]. Hence, a strict follow-up protocol for patients with IgAN is necessary to assess disease severity over time, with extensive investigations and treatment directed at controlling proteinuria and hypertension.

Corticosteroids were the most common drugs prescribed in our study. This is not surprising because of the well-known immunosuppressive effect of corticosteroids. IgAN, being an immune-mediated disease, is effectively controlled with steroids, which explains their widespread use in patients with IgAN. According to a recent study conducted by Wang et al., corticosteroid therapy is likely to be effective and safe for patients with IgAN [27]. Another study suggested that the “legacy effect,” a term that means memory of a therapy that delivers benefits long after the intervention has ended, is heavily implemented in the use of corticosteroids for patients with IgAN; this improves the long-term control of the disease and minimises progression [28]. In another study conducted in 2015, it was reported that the addition of corticosteroids to the management plan was found to slow the rate of decline in renal function and increase the chance of renal survival [29]. Therefore, corticosteroids should be prescribed for patients with IgAN due to their proven effect in controlling disease progression.

Regarding the histopathological features according to the Oxford classification, men had a higher score than women. Additionally, no tubular atrophy (T) or crescents (C) were recorded in female patients, and only one female patient had segmental sclerosis, which might indicate that males usually have worse histopathological findings and rate of renal damage than females, possibly due to the lack of renal protective properties of oestrogen seen in females. As mentioned above, the probable processes underlying the renal protective effect in females appear to be linked to oestrogen [24]. Additionally, two studies found that male IgAN patients had more pathological abnormalities, including a greater rate of tubular atrophy/interstitial fibrosis (T) and arteriosclerosis [11,12]. Furthermore, Wen et al. concluded that male sex was an independent risk factor for poor prognosis [12]. We suggest that more studies should be conducted to assess the sexual differences in clinical and pathological outcomes, as well as to focus on early treatment and control, especially in male patients due to their rapid deterioration and worse prognosis compared to females. Finally, we strongly encourage early biopsy in male patients when IgAN is suspected.

The most prevalent histopathological finding was mesangial proliferation (M1). Several studies have also reported mesangial proliferation (M1) as the most common histopathological finding in IgAN [11,12,21]. This is possible because glomeruli are the first part of nephrons that encounter the altered IgA molecule, leading to inflammation and an increase in mesangial matrix and mesangial hypercellularity. This injury might lead to haematuria, proteinuria, hypertension, and decreased renal clearance [30]. Early detection of haematuria and proteinuria by urinalysis, followed by a renal biopsy to detect mesangial proliferation and appropriate management might decrease inflammation and thus mesangial hypercellularity.

Study limitations include a single study location (tertiary centre in Jeddah, Saudi Arabia), only one ethnic group included, small sample size, poor documentation of data for some patients, especially clinical presentations and laboratory findings. Although this study uses a more recent classification to describe the clinical, pathological, and laboratory features of IgAN in a Saudi cohort, a multicentre and multiethnicity study is recommended to make more relevant comparisons.

## Conclusions

We conclude that there is a difference in the pathological, clinical, and laboratory findings in IgAN between men and women. Men generally have worse laboratory and pathological findings; therefore, we strongly suggest changes in the diagnostic, treatment and follow-up guidelines to accommodate these differences. Moreover, national screening programs focusing on the most common presentations, such as haematuria and hypertension to detect IgAN at an early stage, are important to minimize long-term deterioration in renal function. Finally, we strongly recommend conducting a multicentre and multi-ethnic study to explore the ethnic variability in IgAN in terms of presentations and findings.

## References

[1] Rifai A, Dworkin LD (2008). IgA nephropathy: markers of progression and clues to pathogenesis. Kidney Int.

[2] Al Hussain T, Hussein MH, Al Mana H, Akhtar M (2017). Pathophysiology of IgA nephropathy. Adv Anat Pathol.

[3] Wyatt RJ, Julian BA (2013). IgA nephropathy. N Engl J Med.

[4] Roberts IS, Cook HT, Troyanov S (2009). The Oxford classification of IgA nephropathy: pathology definitions, correlations, and reproducibility. Kidney Int.

[5] AlFaadhel T, Alsuwaida A, Alsaad K (2019). Prevalence and 20-year epidemiological trends of glomerular diseases in the adult Saudi population: a multicenter study. Ann Saudi Med.

[6] Seedat YK, Nathoo BC, Parag KB, Naiker IP, Ramsaroop R (1988). IgA nephropathy in blacks and Indians of Natal. Nephron.

[7] Levy M, Berger J (1988). Worldwide perspective of IgA nephropathy. Am J Kidney Dis.

[8] Schena FP, Nistor I (2018). Epidemiology of IgA nephropathy: a global perspective. Semin Nephrol.

[9] Trimarchi H, Barratt J, Cattran DC (2017). Oxford Classification of IgA nephropathy 2016: an update from the IgA Nephropathy Classification Working Group. Kidney Int.

[10] Nasri H, Mortazavi M, Ghorbani A (2012). Oxford-MEST classification in IgA nephropathy patients: a report from Iran. J Nephropathol.

[11] Deng W, Tan X, Zhou Q (2018). Gender-related differences in clinicopathological characteristics and renal outcomes of Chinese patients with IgA nephropathy. BMC Nephrol.

[12] Wen D, Tang Y, Tan L, Tan J, Chen D, Zhang Y, Qin W (2021). Sex disparities in IgA nephropathy: a retrospective study in Chinese patients. Int Urol Nephrol.

[13] Ghani AA, Al Waheeb S, Al Homoud E, Al Helal B, Hussain N (2011). Clinical and histopathological spectrum of IgA nephropathy in Kuwait. Ann Saudi Med.

[14] Khawajah AQ, Al-Maghrabi J, Kanaan HD, Al-Ghamdi S (2010). IgA nephropathy: a clinicopathologic study from two centers in Saudi Arabia. Saudi J Kidney Dis Transpl.

[15] Tan M, Li W, Zou G, Zhang C, Fang J (2015). Clinicopathological features and outcomes of IgA nephropathy with hematuria and/or minimal proteinuria. Kidney Blood Press Res.

[16] Levey AS, Stevens LA, Schmid CH (2009). A new equation to estimate glomerular filtration rate. Ann Intern Med.

[17] Schwartz GJ, Muñoz A, Schneider MF, Mak RH, Kaskel F, Warady BA, Furth SL (2009). New equations to estimate GFR in children with CKD. J Am Soc Nephrol.

[18] Cattran DC, Coppo R, Cook HT (2009). The Oxford classification of IgA nephropathy: rationale, clinicopathological correlations, and classification. Kidney Int.

[19] Baxmann AC, Ahmed MS, Marques NC, Menon VB, Pereira AB, Kirsztajn GM, Heilberg IP (2008). Influence of muscle mass and physical activity on serum and urinary creatinine and serum cystatin C. Clin J Am Soc Nephrol.

[20] Cattran DC, Reich HN, Beanlands HJ, Miller JA, Scholey JW, Troyanov S (2008). The impact of sex in primary glomerulonephritis. Nephrol Dial Transplant.

[21] Riispere Ž, Laurinavičius A, Kuudeberg A (2016). IgA nephropathy clinicopathologic study following the Oxford classification: progression peculiarities and gender-related differences. Medicina.

[22] Wetzels JF, Kiemeney LA, Swinkels DW, Willems HL, den Heijer M (2007). Age- and gender-specific reference values of estimated GFR in Caucasians: the Nijmegen Biomedical Study. Kidney Int.

[23] Pottel H, Hoste L, Delanaye P, Cavalier E, Martens F (2012). Demystifying ethnic/sex differences in kidney function: Is the difference in (estimating) glomerular filtration rate or in serum creatinine concentration?. Clin Chim Acta.

[24] Garovic VD, August P (2016). Sex differences and renal protection: Keeping in touch with your feminine side. J Am Soc Nephrol.

[25] Lai KN, Tang SC, Schena FP, Novak J, Tomino Y, Fogo AB, Glassock RJ (2016). IgA nephropathy. Nat Rev Dis Primers.

[26] Chen T, Li X, Li Y (2019). Prediction and risk stratification of kidney outcomes in IgA nephropathy. Am J Kidney Dis.

[27] Wang J, Juan C, Huang Q, Zeng C, Liu Z (2013). Corticosteroid therapy in IgA nephropathy with minimal change-like lesions: a single-centre cohort study. Nephrol Dial Transplant.

[28] Coppo R (2013). Is a legacy effect possible in IgA nephropathy?. Nephrol Dial Transplant.

[29] Tesar V, Troyanov S, Bellur S (2015). Corticosteroids in iga nephropathy: a retrospective analysis from the VALIGA study. J Am Soc Nephrol.

[30] Novak J, Julian BA, Mestecky J, Renfrow MB (2012). Glycosylation of IgA1 and pathogenesis of IgA nephropathy. Semin Immunopathol.

